# An Essay on the Yellow Fever, as It Has Occurred in Charleston, Including Its Origin and Progress up to the Present Time

**Published:** 1852-09

**Authors:** 


### BIBLIOGRAPHICAL NOTICES.

---

Art. III.—*An Essay on the Yellow Fever, as it has occurred in Charleston, including its origin and progress up to the present time.* By Thos. Y. Simons, M. D., Port Physician, formerly President of the Medical Society, and Professor of Practice of Physic in the late Medical College of South Carolina, medical adviser and formerly Chairman of the Board of Health, etc. Read before the South Carolina Association, at its Anniversary Meeting, May, 1851. Charleston, S. C. 1851.

In this Essay, Dr. Simons gives, 1st. The history of yellow fever from its first appearance in Charleston to the present time; 2d. The causes which have been supposed to produce the disease; 3d. Describes the former condition of the city, its present condition, and the system of medical police adopted; 4th. Points out the peculiar character of the disease and the diagnosis between it and other fevers of that vicinity; and 5th. Gives a succinct view of the various remedial agents adopted for its cure.

Dr. Simons is a believer in the indigenous character of yellow fever. He believes that while a ship may originate the infection, it cannot propagate it except in an unhealthy atmosphere. He says:

From the statements which I have brought to your view, you will readily perceive, that while I regard it as proper to prevent, as far as practicable, the introduction of yellow fever from other sources, we should not be forgetful to remove all causes which may arise among us to produce the disease. It is a great error to conceive that we receive all the evils of epidemics from other sources, rather than causes among ourselves. The true, and in my opinion, the wise plan, is to attend particularly to internal medical police, with a judicious care as regards introduction of it from other sources. I must in candor say, that I do not believe the yellow fever was ever produced in Charleston from importation; and this is the result of an experience of thirty years as Port Physician, and twenty-four years a member of the Board of Health, many years of which I have been its chairman.

